# Perceived discrimination in *bateyes* of the Dominican Republic: results from the Everyday Discrimination Scale and implications for public health programs

**DOI:** 10.1186/s12889-019-7773-2

**Published:** 2019-11-12

**Authors:** Hunter M. Keys, Gregory S. Noland, Madsen Beau De Rochars, Thomas H. Taylor, Stephen Blount, Manuel Gonzales

**Affiliations:** 1 Department of Anthropology, University of Amsterdam, Building B-REC B 8.01, Nieuwe Achtergracht 166, 1018 WV Amsterdam, The Netherlands; 2 The Carter Center, 453 Freedom Parkway, Atlanta, GA 30307 USA; 3 Health Services Research, Management and Policy Department, University of Florida, 1225 Center Drive, HPNP 3101, Gainesville, FL 32611 USA; 4 Division of Laboratory Systems, Center for Surveillance, Epidemiology, and Laboratory Services, Centers for Disease Control and Prevention, 1600 Clifton Road, Atlanta, GA 30333 USA; 5 Centro de Prevención y Control de Enfermedades transmitidas por Vectores y Zoonosis, Av. Juan Pablo Duarte No. 269, 10301 Santo Domingo, Dominican Republic

**Keywords:** Perceived discrimination, Everyday discrimination scale, Community engagement, Disease elimination, Haiti, Dominican Republic, Malaria, Lymphatic filariasis

## Abstract

**Background:**

Discrimination is a major driver of health disparities among minority groups and can impede the reach of public health programs. In the Dominican Republic, residents of *bateyes*, or agricultural ‘company towns,’ often face barriers to health care. This study examined the extent of perceived discrimination among *batey* populations and places the findings within the context of disease elimination efforts.

**Methods:**

In March—April 2016, a stratified, multi-stage cluster survey that included the 9-item Everyday Discrimination Scale (EDS) was conducted among residents (*n* = 768) of *bateyes* across the Dominican Republic. Exploratory factor analysis, differential item functioning, and linear and logistic regression were used to assess associations between EDS scores, ethnic group status, reasons for discrimination, and healthcare-seeking behavior.

**Results:**

Three ethnic groups were identified in the population: Haitian-born persons (42.5%), Dominican-born persons with Haitian descent (25.5%), and Dominican-born persons without Haitian descent (32.0%). Mean EDS scores (range 0–45) were highest among persons born in Haiti (18.2, 95% confidence interval [CI] = 16.4–20.1), followed by persons with Haitian descent (16.5, 95% CI = 14.9–18.0), and those without Haitian descent (13.3, 95% CI = 12.1–14.5). Higher EDS scores were significantly associated with Haitian birth (β = 6.8, 95% CI = 4.2—9.4; *p* < 0.001) and Haitian descent (β = 6.1, 95% CI = 3.2—9.0; p < 0.001). Most respondents (71.5%) had scores high enough to elicit reasons for their discrimination. Regardless of ethnic group, poverty was a common reason for discrimination, but Haitian-born and Haitian-descended people also attributed discrimination to their origin, documentation status, or skin color. EDS scores were not significantly associated with differences in reported care-seeking for recent fever (β = 1.7, 95% CI = − 1.4—4.9; *p* = 0.278).

**Conclusion:**

Perceived discrimination is common among *batey* residents of all backgrounds but highest among Haitian-born people. Discrimination did not appear to be a primary barrier to care-seeking, suggesting other explanations for reduced care-seeking among Haitian populations. Public health community engagement strategies should avoid exacerbating stigma, build active participation in programs, and work towards community ownership of disease control and elimination goals.

## Background

Perceived discrimination, or perceptions of being treated unfairly among members of minority groups [1], has harmful effects on mental and physical health [1–7] and can impede the reach of public health programs [8–12]. Conceptually, the terms *perceived discrimination* and *discriminatory experiences* refer to the perspectives and experiences of stigmatized groups—those marked by some disqualifying attribute [13, 14]. While *stigma* conjures individual-level attributes or ‘marks,’ *discrimination* and its cognates recall ‘the producers of rejection and exclusion’ [13]—that is, the structural context in which the stigmatized live [15, 16]. As commonly understood, a deeply discrediting attribute, such as race, ethnicity, or sexual orientation, feeds an ideology that construes members with that attribute as inherently inferior or as a threat to the dominant group [14].

From a practical standpoint, studies of perceived discrimination can also inform public health programs that seek to collaborate with disadvantaged social groups. Community engagement refers to the broad set of practices that establish and maintain the human relationships within a public health program, including community members, public health practitioners, outside investigators, and funders [17, 18]. Crucial to any public health program’s success is active community participation, which community engagement strategies try to foster [19]. Thus, an understanding of perceived discrimination can inform engagement strategies seeking to reach stigmatized groups who may harbor feelings of disempowerment or suspicion towards outsiders.

Hispaniola, shared by the Dominican Republic (pop. 10.6 million) and Haiti (pop. 10.8 million) [20], is the only remaining malaria-endemic island in the Caribbean and the site of over 95% of lymphatic filariasis (LF) cases in the Western hemisphere [21]. Both countries have committed to elimination of these mosquito-borne, parasitic diseases, though Haiti bears the greater burden of both diseases. More than 17,000 cases of malaria were reported annually in Haiti from 2013 to 2016, compared to less than 1000 cases annually in the Dominican Republic [22]. Active LF transmission was identified in 88% of Haiti’s communes (districts) and the entire country’s population is considered at-risk and in need of mass drug administration [23]. In the Dominican Republic, LF was restricted to three geographic foci—a small urban focus in the capital Santo Domingo and two larger foci in agricultural areas of the Southwest and the East [24]. Given the higher prevalence of both diseases in Haiti and generally porous border that separates the two countries, it is often assumed that labor migration from Haiti promotes disease transmission in the Dominican Republic [25].

To explore prevalence of malaria and LF in the Dominican Republic, a 2016 nationwide, cross-sectional survey was conducted in Dominican *bateyes*, or agricultural shantytowns reliant on migrant labor from Haiti [26]. Since the late nineteenth century, imported labor from Haiti has been integral to the Dominican economy [27, 28]. Migrant workers settled in *bateyes*, settlement villages adjacent to sugar cane and other plantations throughout the Dominican Republic [28]. Haitian migrants and their descendants have contended with a history of discriminatory practices in the Dominican Republic rooted in legacies of race, class, and nationality [29, 30]. In 2013, the Dominican Constitutional Court issued a verdict, colloquially known as *La Sentencia* (the Sentence), which stripped the right to citizenship of thousands of Dominican-born persons who are primarily of Haitian descent [31, 32]. Lacking documents restricts access to healthcare, education, and job mobility in the country [32].

The goal of this study was to measure perceived discrimination among *batey* residents, elicit reasons for discriminatory experiences, and determine whether perceived discrimination was associated with different ethnic groups using the Everyday Discrimination Scale (EDS), a common measure of perceived discrimination [33]. The EDS displays good reliability (consistency in how people respond to EDS questions) and validity (that it truly measures an underlying discrimination construct) across diverse populations [2, 34–39]. This study was nested within the investigation of malaria and LF prevalence in *bateyes* [40]. While none (0%) of the *batey* study participants were positive for malaria or LF parasites, Haitian-born individuals more frequently reported recent fever and lower levels of care-seeking for the fever compared to Dominican-born *batey* residents [40]. It was hypothesized that the main ethnic groups inhabiting *bateyes* (Haitian-born, Haitian-descended, and non-Haitian-descended people) would all endorse discriminatory experiences but vary in their explanations for them. For example, people with Haitian ancestry were predicted to attribute discriminatory experiences to their nationality or undocumented status. Furthermore, it was thought that perceived discrimination would be associated with reduced care-seeking behavior, particularly among the Haitian-born population. Exploring perceptions and explanations of discrimination can inform public health interventions that seek to reduce barriers to care and generate community-wide support for health programs [41]. Therefore, the practical contribution of this study is to provide a descriptive profile of perceived discrimination in this context and recommendations for public health-oriented community engagement.

## Methods

### Survey design

This study uses data from a nationwide, cross-sectional, multi-stage cluster survey of malaria and LF prevalence among *batey* residents conducted from March—April 2016 near the end of the sugar-cane harvest (*zafra*) [40]. The survey was sponsored by The Carter Center, a US-based not-for-profit health and human rights non-governmental organization, and conducted in collaboration with the Dominican Ministry of Health’s national center for control of tropical diseases (Spanish acronym, CENCET).

**Fig. 1:**
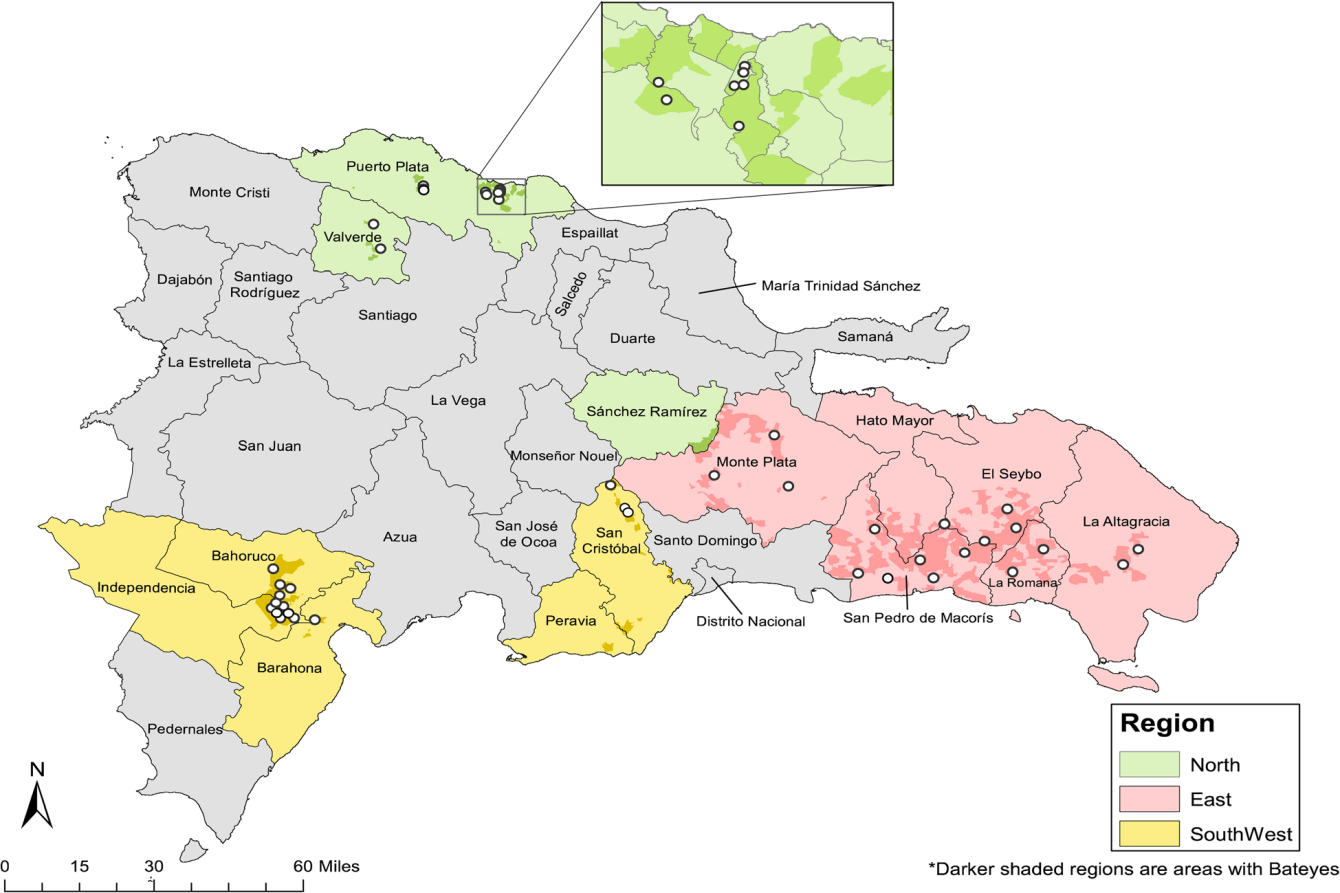
Map illustrating sampled *bateyes* (open circles) in the three geographic strata of *bateyes* in the Dominican Republic

To generate representative disease prevalence estimates from each of the extant agricultural regions in the Dominican Republic, the survey defined three strata: Southwest, East, and North (Fig. 1). Using lists of *bateyes* obtained from a nation-wide *batey* census done in 2012 as a sampling frame [42], a total of 51 clusters (*bateyes*), 17 in each stratum, were selected using systematic (interval) selection from a random start with probability of selection proportional to population size. In each selected cluster, 15 households were systematically selected from a random start using sketch maps prepared by survey teams prior to sampling. For the disease prevalence survey, the target sample size of 482 persons per strata, or 2 persons per household, was sufficient to detect a prevalence of malaria and LF of 5% with absolute precision of ±2.5% at the 95% two-sided significance level with a design effect of 1.5 and a 10% non-response rate. Field teams were comprised of Haitian-born, bilingual (Haitian Kreyòl, Spanish) interviewers.

### Data collection

At each household, self-identified adult (age ≥ 18 years) heads-of-household or his/her spouse were informed about the survey and asked to participate in a household questionnaire. The questionnaire included relevant demographic variables (age, gender, country of birth, language of survey administration, residency status, documentation status, and occupation). Ethnicity was self-reported and based on birth location: 1) Haitian-born; 2) Dominican-born with Haitian descent; and 3) Dominican-born without Haitian descent. The questionnaire also explored recent illness and fever, with follow-up questions for care-seeking. Primary results from these modules, along with parasite diagnostic testing of blood samples collected from the questionnaire respondent and one other randomly selected household member of any age, are reported elsewhere [40]. However, participation in the diagnostic module was not a requirement for participation in the household questionnaire and vice versa—i.e. selected participants could decline either of the two survey components.

The household questionnaire included the original nine-item Everyday Discrimination Scale (EDS) ([33]; see Additional file 2). The EDS elicits responses based on a Likert format to chronic or episodic discriminatory experiences that are essentially minor, followed by suggested reasons for those experiences, such as ancestry, gender, or religion [34]. Answering ‘A few times’ or more to any of the nine EDS items triggered a separate module at the end of the EDS, in which potential reasons for the experience(s) were provided and the participant was asked to what degree the reason accounted for the experience(s). Each given reason was preceded by the question, ‘Considering everything we just talked about, for those things that happened at least a few times or more, how much does it have to do with [reason]?’ The reasons were provided in order of their presumed increasing sensitivity: 1) Poverty or economic problems; 2) Health problems; 3) Lack of education; 4) Language problems (trouble speaking Spanish); 5) Documentation problems; 6) Skin color; and 7) Origin, which was explained to participants as ‘your country of birth’ or ‘where you are from.’ These reasons were selected a priori based on previous ethnographic fieldwork [43] and literature review of Haitian-Dominican relations and the political history of *bateyes*. A 4-point Likert scale was provided for each given reason (with ‘Don’t know’ coded as zero), where 1 = No, [reason] has nothing to do with it; 2 = Yes, a little; 3 = Yes, a lot; and 4 = Yes, very much so.

All survey questions were first translated from English to Spanish and Haitian Kreyòl and discussed in team meetings with native speakers of both languages. Then, the questionnaire was piloted to ensure comprehension and comfort. Survey questions were then back-translated by people not affiliated with the study to compare to the original English. Data were collected electronically using hand-held tablet computers running custom data collection software (Eagle Survey, The Carter Center).

### Analysis

Only questionnaires with completed EDS modules, regardless of participation in the parasite diagnostic module, were included for analysis here. Descriptive statistics were calculated for bivariate associations between demographic factors and ethnic groups. Categorical variables were tested for independence using the adjusted Wald test [44]. Following previous convention [2, 33, 34], an EDS total score was obtained by summation of responses to the nine items, with higher scores indicating higher levels of perceived discrimination. First, mean scores for each EDS item were stratified by ethnic group; higher scores indicated greater frequency of the EDS item happening in daily life. Next, mean EDS total scores were compared between relevant groups based on background variables: ethnic groups, demographics, and care-seeking for recent fever. Cronbach’s alpha (α), a measure of internal consistency ranging from 0 to 1 with higher values indicating that survey items reliably measure the same underlying construct [45], was also obtained within each ethnic group and ranged from 0.81–0.83.

After calculating descriptive statistics of the EDS, exploratory factor analysis (EFA) was conducted to determine dimensionality of the EDS. EFA was performed for each ethnic group to assess whether the nine EDS items represent a single, latent construct or multiple constructs (or ‘factors’). EFA was based on polychoric correlation matrices, which have been shown to be more appropriate for creating common factor models originally based on categorical variables [46]. The factor loadings of each item were rotated to better interpret how strongly each EDS item is correlated with the underlying factor [47]. EFA strongly suggested that the EDS captured a single, latent construct. Within each ethnic group, the eigenvalue of the first factor was high while the remaining eigenvalues for subsequent factors were all less than 1 (Additional file 1: Table S1). Factor loadings of EDS items ranged from 0.44–0.85 across groups, except for the item ‘Less courtesy’ among Dominican-born persons with Haitian descent (0.26).

Following EFA, the EDS was tested for differential item functioning (DIF) using the Mantel-Haenszel approach. Aside from determining if the EDS measures the same latent construct(s) across different groups, it is necessary to assess whether members of each group have equal probabilities of responding similarly to each survey item, after matching to members of a reference group based on EDS total score. There is potential for measurement bias when members of one group appear to respond differently (have a higher or lower probability of responding a certain way) to an item after matching to members of the reference group, which can lead to inflated scores compared to other groups and incorrect inferences about group differences [48]. Separate DIF analyses were undertaken for Haitian-born and Dominican-born persons with Haitian descent compared to Dominican-born persons without Haitian descent (reference group). A final DIF analysis was done in which responses from Dominican-born persons without Haitian descent were compared to both Haitian-born and Haitian-descended people (a combined reference group). Cole’s criterion recommends considering DIF when the odds ratio (OR) of responding to a given item is > 2.0 or < 0.5 [49].

Next, linear regression analyses were done of the following independent variables to assess their significance in predicting EDS total score: age, gender, permanent residency, being documented, completing the survey in Spanish, being employed, seeking care for recent fever, and a categorical variable *Origin* where Haitian-born = 2, Dominican-born with Haitian descent = 1, and Dominican-born without Haitian descent = 0 (reference group). After univariate analyses, significant (*p* < 0.05) variables were then included as covariates in a multivariable linear regression model with EDS total score as the outcome. An interaction term was also included for *Origin* and *Completed the survey in Spanish* to account for its strong pairwise correlation (r = − 0.81, *p* < 0.001).

To explore relationships between each of the 7 potential reasons for discriminatory experiences and each of the 9 EDS items across ethnic groups, a series of 2 × 2 tables were made based on case-exposure pairs (*n* = 63 pairs for each ethnic group). The goal of this stage of analysis was to assess whether there were significant differences in the odds ratios across ethnic groups when members of a group endorsed an EDS item occurring at least ‘A few times’ or more (‘exposure’) and attributing a given reason as having ‘A lot’ or ‘Very much’ to do with that EDS item (‘case’). Responses to each reason were re-coded as a binary outcome (‘case’) where 1 = [reason] has ‘A lot’ or ‘Very much’ to do with the EDS item and 0 = ‘A little,’ ‘Nothing to do with it’ or ‘Don’t know.’ Similarly, each EDS item was re-coded as a binary predictor (‘exposure’) where 1 = [EDS item] was said to happen ‘A few times’ or more and 0 = ‘Almost never’, ‘Never,’ or ‘Don’t know.’ Logistic models were then made for each case-exposure pair of reason and EDS item, stratified by ethnic group. Significant differences in odds ratios were based on the Breslow-Day statistic, which tests for homogeneity of odds ratios across stratified groups [50].

All statistical analyses were done in Stata v.14.2 (College Station, TX, USA). Population estimates, 95% confidence intervals (CI), and linear and logistic regressions were calculated using Stata’s *svy* routine with sampling weights to account for sampling weights, clustering effects, and stratification. Polychoric matrices for EFA were made using the user-written Stata command *polychoric* and assessment of DIF was done using the *difmh* command. *P*-values less than 0.05 were considered statistically significant.

## Results

A total of 780 individuals participated in the household survey. The majority identified as the head-of-household (84.3%). A response rate could not be calculated because field personnel did not record rejection or absenteeism. Excluding individuals with missing EDS responses, a total of 768 completed questionnaires were included in the final analysis.

### Demographics

Table 1 summarizes population estimates of demographic characteristics, stratified by ethnic group. Haitian-born persons comprised 42.5% of the *batey* population, Dominican-born persons with Haitian descent 25.5%, and Dominican-born persons without Haitian descent 32.0%. There were significant differences in the composition of ethnic groups within each stratum: most Haitian-born persons were in the East (51.1%), most of those with Haitian descent were in the Southwest (50.3%), and most Dominican-born without Haitian descent were in the North (62%) (*p* < 0.001).

**Table 1:** Characteristics of *batey* residents stratified by ethnic group, Dominican Republic, 2016, (*n* = 768; population-level estimates shown)

Characteristic	Haitian-born	Dominican-born, Haitian descent	Dominican-born, no Haitian descent	*p* adj. Wald test^*^
% (95% CI)	42.5% (32.0—53.7)	25.5% (21.3—30.1)	32.0% (21.8—44.4)	
*Region*				
Southwest	18.7 (12.2—27.4)	50.3 (36.0—64.5)	31.1 (17.9—48.3)	< 0.001^*^
North	24.4 (10.1—48.2)	13.6 (7.4—23.6)	62.0 (34.9—83.3)	
East	51.1 (37.6—64.5)	23.1 (19.4—27.2)	25.8 (15.5—39.8)	
Age in years, mean (SE)	43.7 (2.5)	41.3 (1.6)	50.0 (2.5)	0.024^*^
Female	39.2 (29.0—50.5)	68.8 (59.3—77.0)	62.6 (52.3—71.8)	0.001^*^
Completed survey in Spanish	3.6 (1.3—9.4)	34.5 (24.8—45.8)	97.7 (93.6—99.2)	< 0.001^*^
Documented	73.2^♦^ (64.4—80.6)	85.8 (74.3—92.7)	99.5 (96.9—99.9)	< 0.001^*^
Permanent resident	76.8^♦^ (67.8—83.9)	85.9^♦^ (79.2—90.6)	95.2 (90.9—97.5)	0.011^*^
Unemployed	27.1 (19.1—37.1)	39.0 (28.2—51.0)	46.5 (39.7—53.4)	0.001^*^

*Abbreviation*: *CI* Confidence interval, *SE* Standard error

^*^Indicates statistically significant (*p* < 0.05)

^♦^Missing = 1

There were fewer female Haitian-born residents (39.2%) compared to the other two groups (*p* = 0.001), suggesting an active male-dominated migrant workforce. Unsurprisingly, most (96.4%) Haitian-born residents undertook the survey in Kreyòl, whereas 34.5% of Dominican-born with Haitian descent and 97.7% of those without Haitian descent completed the survey in Spanish (*p* < 0.001). Third, most residents reported having some form of official documentation, though proportions were significantly different across ethnic groups: 73.2% of Haitian-born were documented, compared to 85.8% of Dominican-born with Haitian descent and 99.5% of Dominican-born without Haitian descent (*p* < 0.001). Fourth, most of those living in *bateyes* were permanent residents (having spent at least 9 consecutive months in the *batey* at any point since the year 2013), with an upward trend in permanent residency across Haitian-born (76.8%), Dominican-born with Haitian descent (85.9%), and Dominican-born without Haitian descent (95.2%) (*p* = 0.011). Finally, proportionally more Dominican-born persons without Haitian descent were unemployed (46.5%) compared to Dominican-born with Haitian descent (39.0%) and Haitian-born persons (27.1%) (*p* = 0.001).

### EDS characteristics

Table 2 displays total mean scores and mean scores of each EDS item, stratified by ethnic group. Total mean EDS scores were highest among Haitian-born persons (18.2) followed by Dominican-born with Haitian-descent (16.5) and lastly Dominican-born without Haitian descent (13.3) (*p* < 0.001). Seven of the nine individual EDS items were significantly different across groups, with Haitian-born individuals tending to have higher mean scores followed by persons of Haitian descent and lastly those without Haitian descent. Mean scores of two EDS items were not significantly different across groups: ‘Not smart’ and ‘Act afraid.’

**Table 2:** Mean scores of Everyday Discrimination Scale (EDS) items, stratified by ethnic group, Dominican Republic, 2016 (*n* = 768; population-level estimates shown)

	Haitian-born	Dominican-born, Haitian descent	Dominican-born, no Haitian descent	*p* adj. Wald test^*^
9-item EDS^a^, mean (95%CI)	18.2 (16.4–20.1)	16.5 (14.9–18.0)	13.3 (12.1–14.5)	< 0.001^*^
Cronbach’s alpha, EDS	0.83	0.81	0.82	–
EDS item^b^, mean (95% CI) *Haitian Kreyòl translation* *Spanish translation*				
People treat you with less courtesy than others. *Moun yo bay’w mwens atensyon (afeksyon) ke sa yo bay yon lòt moun.* *Se siente usted tratada con menos cortesía en comparación con otras personas.*	2.02 (1.88–2.17)	1.79 (1.57–2.01)	1.47 (1.29–1.65)	< 0.001^*^
People treat you with less respect than others *Moun yo bay’w mwens respe ke sa yo bay yon lòt moun.* *Las gentes le brindan menos respeto en comparación con otras personas.*	2.23 (1.96–2.49)	2.17 (1.93–2.42)	1.66 (1.43–1.89)	< 0.001^*^
You receive poorer service than other people in stores, bodegas, markets, or in the street *Nan boutik, magazen, mache, bodega, oubyen nan lari, yo trete’w pi mal ke lòt moun.* *En las tiendas, almacenes, mercado, bodegas, o en la calle, le tratan con peor servicio que los demás.*	1.94 (1.70–2.17)	1.51 (1.26–1.75)	1.31 (1.18–1.44)	< 0.001^*^
People act as if they think you are not smart *Moun yo kompote yo kom si yo kwe ou pa entelijan.* *La gente se comporta como si usted no fuera una persona inteligente.*	1.95 (1.72–2.17)	1.57 (1.30–1.83)	1.55 (1.34–1.76)	0.088
People act as if they are afraid of you *Moun yo kompote yo kom si yo pè’w.* *La gente se comporta con miedo hacia usted.*	1.48 (1.32–1.64)	1.53 (1.25–1.81)	1.26 (1.08–1.45)	0.138
People act as if they think you are dishonest and do not trust you *Moun yo panse ke ou pa onèt ou byen kom si yo pa fe’w konfyans.* *La gente se comporta como si usted fuera deshonesto/a y no le tienen confianza.*	1.61 (1.31–1.91)	1.61 (1.37–1.85)	1.22 (1.13–1.32)	0.007^*^
People act as if they’re better than you *Moun yo kompote yo kom si yo panse yo pi bon pase ou.* *La gente se comporta como si fueran mejor que usted.*	2.74 (2.37–3.12)	2.69 (2.43–2.96)	2.0 (1.59–2.38)	0.013^*^
People call you names or make fun of you *Moun yo moke ou ou byen bay ou vye nom.* *La gente se burla de usted.*	2.08 (1.74–2.42)	1.85 (1.58–2.12)	1.43 (1.20–1.66)	0.017^*^
You feel threatened by other people *Moun yo konn menase ou.* *Se siente amenazado/a por otras personas.*	2.19 (1.87–2.51)	1.75 (1.42–2.08)	1.36 (1.19–1.54)	0.001^*^

*Abbreviation*: *EDS* Everyday Discrimination Scale, *CI* Confidence interval

^a^Range = 0–45; higher scores indicate higher reports of everyday discrimination

^b^Range = 0–5; higher scores indicate greater frequency of EDS item

^*^ Indicates statistically significant (*p* < 0.05)

The differential item functioning (DIF) analysis revealed two EDS items with meaningful DIF: ‘Not smart’ and ‘You feel threatened.’ The odds of answering ‘A few times’ or more to ‘People act like you are not smart’ were higher among Dominican-born without Haitian descent (*p* = 0.016) compared to both Haitian-born and Haitian-descended people. Conversely, the odds of answering ‘A few times’ or more to the item ‘You feel threatened’ was significantly higher among Haitian-born residents (*p* = 0.001) compared to Dominican-born persons without Haitian descent, with a similar but not statistically significant trend (*p* = 0.096) observed for Dominican-born individuals with Haitian descent. The remaining seven EDS items were not significant in DIF analyses, indicating that irrespective of ethnic group status, those items elicited similar responses among participants matched on EDS total score.

### EDS: linear regression analysis

Univariate linear regression sought to identify independent variables associated with EDS total score. Male gender, being employed, Haitian birth, and Haitian descent were all significantly associated with higher EDS total scores (Table 3). Conversely, completing the survey in Spanish was significantly associated with a lower EDS total score. Age, permanent residency, being documented, and seeking care for recent fever were not significantly associated with EDS total score.

**Table 3:** Univariate linear regression of total EDS score, Dominican Republic, 2016 (*n* = 768)

Variable	β	SE	95% CI	*p*
Age	0.03	0.02	−0.1—0.1	0.196
Male	1.8	0.7	0.4—3.2	0.012^*^
Permanent resident	−1.0	1.6	−4.3—2.3	0.551
Documented	−1.2	1.0	−3.2—0.8	0.221
Completed survey in Spanish	−4.2	1.1	−6.4—-2.0	< 0.001^*^
Employed	1.7	0.7	0.2—3.2	0.027^*^
[*If had fever in previous 2 weeks*]: Sought care for fever	1.7	1.6	−1.4—4.9	0.278
Origin^a^				
Dominican-born, Haitian descent	3.2	1.0	1.2—5.3	0.003^*^
Haitian birth	5.0	1.2	2.6—7.3	< 0.001^*^

*Abbreviations*: *SE* Standard error, *CI* Confidence interval

^a^Reference group: Dominican-born, no Haitian descent

^*^Indicates statistically significant (*p* < 0.05)

Independent variables found to be significant in the univariate analysis were then used to fit a multivariable linear regression model of EDS total score (Table 4). The overall regression model was significant (*p* < 0.001). Adjusting for other variables in the model, those born in the Dominican Republic with Haitian descent had a 6.1-point increase in their EDS scores (95% CI = 3.2—9.0; *p* < 0.001) and Haitian-born persons had a 6.8-point increase (95% CI = 4.2—9.4; p < 0.001). Furthermore, Dominican-born persons with Haitian descent who completed the survey in Spanish had a − 4.5-point *decrease* in EDS total score (95% CI: − 8.4— -0.7; *p* = 0.022). All other covariates were not significantly associated with EDS total score after adjusting for other variables in the model.

**Table 4:** Multivariable linear regression of total EDS score, Dominican Republic, 2016 (*n* = 768)

Variable	Model significance: *p* < 0.001			
	β	SE	95% CI	*p*
Origin^a^				
Dominican-born, Haitian descent	6.1	1.4	3.2—9.0	< 0.001^*^
Haitian birth	6.8	1.3	4.2—9.4	< 0.001^*^
Completed survey in Spanish	2.1	1.2	−0.4—4.6	0.097
Origin*Completed survey in Spanish				
Haitian descent * Spanish	−4.5	1.9	−8.4— -0.7	0.022^*^
Haitian-born * Spanish	−4.7	2.9	−10.7—1.2	0.116
Male	0.8	0.7	−0.7—2.2	0.313
Employed	0.8	0.8	−0.8—2.3	0.308

*Abbreviations*: *SE* Standard error, *CI* Confidence interval

^a^Reference group: Dominican-born, no Haitian descent

^*^Indicates statistically significant (*p* < 0.05)

### Reasons for EDS experiences

Most individuals (71.5%) were found to experience any EDS item at least ‘A few times’ or more. This occurred most frequently among Haitian-born residents (81.7%) and Dominican-born of Haitian descent (76.2%), but also among more than half (54.4%) of those born in the Dominican Republic without Haitian descent (*p* = 0.005). Among those who answered ‘A few times’ or more to any EDS item, significant differences between ethnic groups were noted in the mean scores of the seven given reasons for EDS experiences (Table 5). First, mean scores of ‘Health problems’ as a reason for any EDS item were not significantly different across groups (*p* = 0.115); given that mean scores for this reason were low compared to mean scores of other reasons, all groups seemed to agree that ‘Health problems’ were not particularly explanatory for discriminatory experiences. However, ‘Poverty/economic problems’ seemed to be especially meaningful in explaining why EDS experiences were said to occur: within each ethnic group, mean scores of ‘Poverty/economic problems’ were greater than all other reasons, although significant differences were noted across ethnic groups (*p* < 0.001). Certain reasons appeared relevant for Haitian-born and Dominican-born persons with Haitian descent. For example, mean scores of ‘Documentation problems’ were 1.98 among Haitian-born persons and 1.32 among Haitian-descended people, yet 1.03 among persons without Haitian descent (p < 0.001). ‘Skin color’ was another reason with notable differences across groups: among Haitian-born persons, mean score was 2.27 and 1.92 among Haitian-descended people, compared to 1.32 among those without Haitian descent (p < 0.001). The reasons that appear more relevant for Dominican-born people without Haitian descent were ‘Poverty/economic problems’ and ‘Lack of education.’

**Table 5:** Degree to which reasons account for EDS experiences, Dominican Republic, 2016 (*n* = 431; population-level estimates shown)

	Haitian-born	Dominican-born, Haitian descent	Dominican-born, no Haitian descent	*p* adj. Wald
Score above threshold to elicit reasons	81.7%	76.2%	54.4%	0.005^*^
Given reason, mean (95%CI); range 0–4				
Poverty/economic problems	2.89 (2.68–3.12)	2.47 (2.20–2.74)	2.12 (1.82–2.41)	< 0.001^*^
Health problems	1.43 (1.27–1.59)	1.17 (0.97–1.37)	1.46 (1.25–1.67)	0.115
Lack of education	2.26 (2.07–2.45)	1.78 (1.50–2.07)	1.55 (1.25–1.85)	< 0.001^*^
Problems speaking Spanish	1.77 (1.50–2.03)	1.24 (1.05–1.43)	1.26 (1.13–1.39)	< 0.001^*^
Documentation problems	1.98 (1.60–2.37)	1.32 (1.15–1.50)	1.03 (0.96–1.10)	< 0.001^*^
Skin color	2.27 (2.10–2.44)	1.92 (1.68–2.15)	1.32 (1.11–1.52)	< 0.001^*^
Origin	2.58 (2.34–2.81)	1.58 (1.38–1.78)	1.15 (1.02–1.28)	< 0.001^*^

*Abbreviations*: *CI* Confidence interval

^*^Indicates statistically significant (*p* < 0.05)

Breslow-Day tests of homogeneity predicted the odds of a reason having ‘A lot’ or ‘Very much’ to do with an EDS item occurring at least ‘A few times’ or more. Of all the pairings between each EDS item and each given reason (*n* = 63 pairs for each ethnic group), only 2 item-reason pairs were significant: the odds of endorsing poverty as having ‘A lot’ or ‘Very much’ to do with being treated with less respect were approximately 5 times higher among both Haitian-born (OR = 4.5; 95% CI = 2.2—9.1) and Dominican-born persons with Haitian descent (OR = 5.1; 95% CI = 2.3—11.3) (*p* = 0.021). Second, Dominican-born persons with Haitian descent were 5.7 times more likely (95% CI = 1.3—33.7) to attribute being called names or insulted to documentation problems (*p* = 0.029).

## Discussion

Most people living in *bateyes* of the Dominican Republic are permanent residents, rather than migrants, and appear to regularly experience some form of interpersonal discrimination that they interpret as a result of poverty. Haitian birth and Haitian descent were strongly associated with high EDS scores; in addition to poverty, members of those ethnic groups also linked discrimination to their origin, documentation status, or skin color. EDS scores were not significantly associated with care-seeking for recent fever, nor were discriminatory experiences understood to occur because of health problems or disease.

As anticipated, perceived discrimination was highest among persons of Haitian ancestry—including both Haitian-born and Haitian-descended people born in the Dominican Republic. In contrast to those born in the Dominican Republic without Haitian descent—whose interpersonal experiences may be subtle, such as being treated as though they are not smart—Haitian-born and Haitian-descended people appear to experience more overt forms of discrimination, like feeling threatened or being called names. Additionally, Haitian-born and Haitian-descended people attributed discriminatory experiences to individual-level ‘marks’ that have been historically denigrated in Dominican society: skin color and origin [30]. Interestingly, poverty and documentation problems were linked to specific EDS experiences (being treated with less respect and being called names, respectively). Poverty likely serves as an index of social status and may be seen as a failure to meet social expectations [51]. That Dominican-born persons with Haitian descent were more likely to attribute being called names to their documentation problems potentially indicates how institutional decisions like the 2013 *Sentencia*, which disproportionately affected this group by taking away their right to citizenship [32], plays out in daily life. At the same time, it is notable that Spanish language capacity appeared to have a protective effect against perceived discrimination among members of this group; it is likely that linguistic differences also signal in- and out-group status.

Persons born in the Dominican Republic without Haitian descent also linked poverty and interpersonal discrimination. There are some possible explanations for this finding. First, data from the linked head-of-household survey found that unemployment was highest among Dominican-born without Haitian descent (46.5%) [40]. In the context of EDS module, ‘Lack of education’ was the second highest reason for discrimination (after ‘Poverty/economic problems’) for that ethnic group, while those without Haitian ancestry were twice as likely to endorse the EDS item ‘People act like you are not smart’ even after matching to Haitian-born and Dominican-born, Haitian-descended people based on EDS total score. These findings suggest that in *bateyes*, Dominican-born persons without Haitian descent link their discriminatory experiences to having little economic or educational opportunities and possibly feeling shut out from a job market that prefers imported, Haitian labor. Aside from dynamics of labor migration from Haiti, it is also possible that economies in and around *bateyes* simply rely on a younger workforce, as the mean age of Dominican-born participants without Haitian descent was greater than Haitian-born and Haitian-descended people. Still, poverty itself seems to be stigmatizing for all those living in *bateyes*. In-depth, qualitative investigations could help tease apart how reasons for stigma (poverty, lack of education, skin color, or origin) are understood among *batey* residents.

Distinctions of economic position, documentation status, language skills, or ethnic origin constitute symbolic marks that shape a sense of place of both self and others [52]. While these marks provide substance for cognitive and evaluative beliefs about social positions, they result from material and social processes [53]: economic exploitation as well as historical ideologies of race and nationality help to reinforce social hierarchies that can be both objectively differentiated (whether by income, language capacity, documentation status, or origin) as well as—and perhaps more importantly—*perceived by* those in a local world, ‘those agents who possess the code, the classificatory schemes necessary to understand their social meaning’ [52]. These classificatory schemes can be subtle or misrecognized [54]. Given the history of *anti-haitianismo* (anti-Haitianism) in the Dominican Republic [55], it may seem obvious that Haitian-born and Dominican-born, Haitian-descended people might suffer more interpersonal discrimination compared to Dominican-born persons without Haitian descent. Still, it is striking that so many—within all ethnic groups—linked their experiences to economic precariousness. Poverty, and class struggle more broadly, may figure into experiences and interpretations of everyday discrimination more so than such marks as skin color or Haitian origin per se. Of course, these elements can and do layer upon each other, or ‘conjugate,’ to compound the suffering of those who may bear more than one mark alone [53].

### Implications for community engagement and disease elimination

This study found high levels of perceived discrimination among Haitian-born individuals, who have historically been implicated in malaria and LF transmission in the Dominican Republic [25, 56]. However, the linked epidemiological survey did not detect any malaria or LF parasite-positive individuals [40]. The apparent interruption of transmission in *bateyes* possibly reflects effective active surveillance for malaria and successful community engagement for LF [57, 58], which must continue until island-wide elimination is achieved. While perceived discrimination itself did not appear to be significantly associated with reduced care-seeking, the discriminatory experiences endorsed by *batey* residents point to an overall sense of disempowerment as well as structural obstacles, especially poverty, that challenge active community participation and ownership of disease elimination goals.

Given higher prevalence of vector-borne disease in Haiti and history of discrimination against this population in Dominican society, public health programs that explicitly link the Haitian-born population to vector-borne disease can exacerbate stigma and blame [10, 59]. Most participants in this survey did not attribute their discriminatory experiences to health conditions, however, and malaria itself is rarely stigmatizing. Still, linking a specific disease to a particular ethnic or social group, no matter how implicitly, can fray social relations in a community [60]. For example, as cholera spread from Haiti to the Dominican Republic, public health messages that emphasized individual-level, preventative behaviors were incorporated into narratives that ignored structural problems of healthcare access and sanitation coverage and instead cast Haitians as directly responsible for the epidemic [59].

A more positive example comes from the country’s LF elimination program, which has fostered active participation in elimination activities by expressly avoiding the issue as one caused by Haitian migrants but rather one of collective responsibility [57]. It is important for any elimination message to emphasize the structural backdrop against which disease occurs, in addition to individual-level, preventative behaviors. While encouraging people to use bed nets, reduce mosquito habitats, and seek care for fever, for example, it will be just as helpful to develop messages that draw on themes of community cooperation. One approach to developing an appropriate public health discourse surrounding malaria and LF would be to collaborate with the communities themselves, which are frequently accustomed to external agencies and organizations initiating health projects. As one study in Haiti demonstrated, even misuse of certain terminology for program participation and purpose can have unintended consequences [61]. Community dialogue, rather than top-down, educative ‘talks,’ can allow community members to have more active roles in shaping health messages [62].

This study also sheds additional light onto the complex entanglement of poverty, perceived discrimination, and risk for disease. It is easily understood that poverty is bad for health, but narrowly focusing on material deprivation (such as lack of clean water or mosquito nets) to control infectious disease has its shortcomings [63]. The cumulative effect of perceived discrimination, social exclusion, and psychological distress contributes to a chronically activated stress response that leaves the body more vulnerable to disease [5, 64, 65]. Stigma-related stress also harms self-esteem and leads to feelings of disempowerment and loss of control in one’s life [66–68].

While perceived discrimination was not significantly associated with care-seeking in this study, it was still intimately related to economic hardship. These relationships among poverty, discrimination, and the likely toll on one’s sense of control in life are relevant for disease programs. For example, in the prevalence survey, the most commonly endorsed reason for not seeking care for fever was that, ‘The illness was not serious enough’ [40]. In the early course of a febrile illness like malaria or LF, more pressing needs—such as economic demands—likely lead people to defer care. This is especially true in low-transmission settings, where the rarity of the disease can lead people to deprioritize treatment and prevention [19]. Compounding matters, previous studies with this population have found an internalized sense of devaluation as well as feeling unable to change life’s circumstances [43, 59]. Such ‘hidden distress’ can in turn limit the degree to which people participate in, and ultimately take ownership of, a community health program [9, 10]. Public health programs can too easily conceptualize people as individual agents with control in their lives; based on these and other findings, eliminating malaria and LF will require more than simply encouraging people to seek care when ill.

As such, community engagement strategies should strive to align the goal of elimination with the day-to-day concerns of community members. A starting point would be to elicit the community’s concerns and identify how malaria and LF elimination activities may (however partially) overlap with them. For example, generating interest and participation in community-level surveillance, or strengthening human relationships between communities and the health system, would all help to build resilience and human capacity in the face of more engrained problems of poverty and discrimination. Community-level workshops between health program staff and community members could incorporate cross-cutting interventions to reduce stigma and perceived discrimination, such as peer counseling, skills building, self-help groups, and micro-credit instruments, all of which can positively impact health [12].

Finally, malaria and LF elimination activities could be part of a larger, community-driven push for human rights, including the right to health and basic services [69]. Again, the country’s LF elimination program is an example of how, despite decades of increasingly hostile immigration and citizenship restrictions, significant progress was made in reducing LF in *bateyes* [57]. This success has been attributed to an approach that favored going through local authority structures (*juntas de vecinos*, or neighborhood associations) and building on existing resources—mainly, local volunteers recruited from within *bateyes*. Such an approach could be considered in remaining areas of malaria transmission.

## Limitations

There are important limitations to this study. First, while the EDS has been adapted for use in cross-cultural contexts [2, 34], it was originally developed among African-Americans in the United States [33]. Consequently, the interpersonal experiences comprising EDS survey items were developed within a specific cultural milieu and may not fully capture experiences of those living in *bateyes* of the Dominican Republic. While exploratory factor analysis of the EDS in this study revealed a unidimensional construct, more in-depth, qualitative research could explore other discriminatory experiences relevant to the lives of *batey* residents. Furthermore, the two EDS items displaying differential functioning (‘People act like you are not smart’ and ‘You feel threatened’) may have introduced measurement bias to inflate scores among Dominican-born without Haitian descent and Haitian-born and Haitian-descended individuals, respectively. These items deserve ethnographic exploration to ascertain why members of certain ethnic groups appear to more readily endorse those items even after matching to members of a reference group. Additionally, qualitative research could help to tease out the specific circumstances under which EDS experiences occur.

Because the survey was conducted in two languages (Spanish, Haitian Kreyòl), there is the potential for measurement bias in how certain questions were asked in their respective languages. While the survey team contacted participants on weekend evenings (when most residents were said to be home), some residents were no doubt missed, potentially introducing additional bias. The study relied on a priori reasons for discrimination based on the authors’ previous fieldwork in this context and literature review. However, there could be additional reasons for discriminatory experiences that were missed by the study. Although consistent with previous studies [2, 33], this study converted Likert responses into a summed, continuous outcome variable (EDS total score). Summation of Likert responses into a presumably continuous variable assumes that the categories of the response (never, almost never, a few times, etc.) are equally distant from each other regardless of item. Despite this limitation, conversion of responses to EDS items into a continuous outcome variable was justifiable for several reasons. Within each ethnic group, the 9-item EDS displayed high internal consistency, unidimensionality, and near-normal distribution statistics: for Haitian-born, EDS total score skewness = 1.1, kurtosis = 4.2; for Dominican-born with Haitian descent skewness = 1.4, kurtosis = 5.4; for Dominican-born without Haitian descent, skewness = 2.0, kurtosis = 8.2 (skewness > 2 or kurtosis > 7 elicit concern for violation; see [70]), supporting the assumption that a summed, continuous outcome provided a reasonable measure of perceived discrimination.

## Conclusion

Perceived discrimination, a social stressor with adverse physical and mental health effects, is commonly experienced among residents of *bateyes* in the Dominican Republic. Haitian ancestry was significantly associated with higher levels of perceived discrimination. Participants tended to link discrimination to markers of inequality, such as poverty, skin color, documentation status, lack of education, and ethnic origin. The stigma of poverty appears to affect the lives of many, regardless of ethnic group. While there is little to no active transmission of malaria and LF in *bateyes* and perceived discrimination per se does not appear to impede care-seeking, active community participation will be essential for ongoing surveillance and elimination efforts. Consequently, community engagement strategies can draw on these findings to contextualize disease elimination goals with people’s everyday concerns.

## Supplementary information

**Additional file 1: Table S1.** Factor loadings after oblique rotation of 9-item EDS using polychoric matrices, stratified by ethnic group, Dominican Republic, 2016. Results of factor analysis of 9-item Everyday Discrimination Scale among three ethnic groups (Haitian-born, Dominican-born without Haitian descent, and Dominican-born without Haitian descent).

**Additional file 2: **EDS English version for DR. Everyday Discrimination Scale used in 2016 *batey* survey. Nine-item Everyday Discrimination Scale and provided reasons for discriminatory experiences adapted for use in survey.

## Data Availability

The dataset used and analyzed in this study is available from the corresponding author on reasonable request.

## Abbreviations

CI: Confidence interval
DIF: Differential item functioning
EDS: Everyday Discrimination Scale
EFA: Exploratory factor analysis
LF: Lymphatic filariasis
OR: Odds ratio
