# Supplementary material for: Perceived discrimination in bateyes of the Dominican Republic: results from the Everyday Discrimination Scale and implications for public health programs
Source: BMC Public Health. 2019 Nov 12;19:1513. doi: 10.1186/s12889-019-7773-2 (PMC6852895; doi:10.1186/s12889-019-7773-2)
Supplement: Supplementary file 1 — Additional file 1: Table S1. Factor loadings after oblique rotation of 9-item EDS using polychoric matrices, stratified by ethnic group, Dominican Republic, 2016. Results of factor analysis of 9-item Everyday Discrimination Scale among three ethnic groups (Haitian-born, Dominican-born without Haitian descent, and Dominican-born without Haitian descent). [file 12889_2019_7773_MOESM1_ESM.docx]

**Table S1: Factor loadings after oblique rotation of 9-item EDS using polychoric matrices, stratified by ethnic group, Dominican Republic, 2016.**

|  | **Haitian-born**  n=256 | **Dominican-born, Haitian descent**  n=224 | **Dominican-born, no Haitian descent**  n=288 | **Overall**  N=768 |
| --- | --- | --- | --- | --- |
| **Factor**  **Eigen value**  **Proportion** | 1  3.51  81.1% | 1  3.45  70.6% | 1  4.10  85.1% | 1  3.54  91.1% |
| **EDS Item** |  |  |  |  |
| Less courtesy | 0.54 | 0.26 | 0.67 | 0.52 |
| Less respect | 0.81 | 0.80 | 0.73 | 0.80 |
| Poorer service | 0.68 | 0.67 | 0.62 | 0.68 |
| People act as if they think you are not smart | 0.44 | 0.53 | 0.45 | 0.45 |
| People act as if they are afraid of you | 0.56 | 0.61 | 0.85 | 0.57 |
| People act as if they think you are dishonest and do not trust you | 0.58 | 0.54 | 0.71 | 0.57 |
| People act as if they’re better than you | 0.66 | 0.57 | 0.77 | 0.67 |
| Called names/harassed by others | 0.56 | 0.68 | 0.70 | 0.63 |
| Threatened by others | 0.70 | 0.75 | 0.49 | 0.68 |
