# Supplementary material for: Perceived discrimination in bateyes of the Dominican Republic: results from the Everyday Discrimination Scale and implications for public health programs
Source: BMC Public Health. 2019 Nov 12;19:1513. doi: 10.1186/s12889-019-7773-2 (PMC6852895; doi:10.1186/s12889-019-7773-2)
Supplement: Supplementary file 2 — Additional file 2: EDS English version for DR. Everyday Discrimination Scale used in 2016 batey survey. Nine-item Everyday Discrimination Scale and provided reasons for discriminatory experiences adapted for use in survey. [file 12889_2019_7773_MOESM2_ESM.docx]

**Everyday Discrimination Scale used in 2016 *batey* survey**

*Main question:* In your life, how often do any of the following things happen to you?

1. People treat you with less courtesy than they treat other people.

Always **5**

Almost always **4**

A few times **3**

Almost never **2**

Never **1**

Don’t know **0**

1. You are treated with less respect than other people are.

Always **5**

Almost always **4**

A few times **3**

Almost never **2**

Never **1**

Don’t know **0**

1. You receive poorer service than other people in stores, bodegas, markets, or in the street.

Always **5**

Almost always **4**

A few times **3**

Almost never **2**

Never **1**

Don’t know **0**

1. People act as if they think you are not smart.

Always **5**

Almost always **4**

A few times **3**

Almost never **2**

Never **1**

Don’t know **0**

1. People act as if they are afraid of you.

Always **5**

Almost always **4**

A few times **3**

Almost never **2**

Never **1**

Don’t know **0**

1. People act as if they think you are dishonest and do not trust you.

Always **5**

Almost always **4**

A few times **3**

Almost never **2**

Never **1**

Don’t know **0**

1. People act as if they’re better than you are.

Always **5**

Almost always **4**

A few times **3**

Almost never **2**

Never **1**

Don’t know **0**

1. People call you names or make fun of you.

Always **5**

Almost always **4**

A few times **3**

Almost never **2**

Never **1**

Don’t know **0**

1. You feel threatened by other people.

Always **5**

Almost always **4**

A few times **3**

Almost never **2**

Never **1**

Don’t know **0**

**Reasons for EDS experiences module**

1. Considering everything we just talked about, for those things that happened frequently to you (that is to say, more than a few times), does it have to do with economic problems or poverty?

THIS QUESTION ONLY APPLIES IF ANY RESPONSE TO QUESTIONS 1-9 WAS 3 OR MORE (A FEW TIMES OR MORE)

Yes, very much so **4**

Yes, a lot **3**

Yes, a little **2**

No, nothing [to do with it] **1**

Don’t know **0**

1. Considering everything we just talked about, for those things that happened frequently to you (that is to say, more than a few times), does it have to do with health problems?

THIS QUESTION ONLY APPLIES IF ANY RESPONSE TO QUESTIONS 1-9 WAS 3 OR MORE (A FEW TIMES OR MORE)

Yes, very much so **4**

Yes, a lot **3**

Yes, a little **2**

No, nothing [to do with it] **1**

Don’t know **0**

11a. If you say yes, what is the illness or health problem? *Write name of illness or health problem ____*

1. Considering everything we just talked about, for those things that happened frequently to you (that is to say, more than several times per year), does it have to do with your level of education?

THIS QUESTION ONLY APPLIES IF ANY RESPONSE TO QUESTIONS 1-9 WAS 3 OR MORE (A FEW TIMES OR MORE)

Yes, very much so **4**

Yes, a lot **3**

Yes, a little **2**

No, nothing [to do with it] **1**

Don’t know **0**

1. Considering everything we just talked about, for those things that happened frequently to you (that is to say, more than a few times), does it have to do with not being able to speak Spanish?

THIS QUESTION ONLY APPLIES IF ANY RESPONSE TO QUESTIONS 1-9 WAS 3 OR MORE (A FEW TIMES OR MORE)

Yes, very much so **4**

Yes, a lot **3**

Yes, a little **2**

No, nothing [to do with it] **1**

Don’t know **0**

1. Considering everything we just talked about, for those things that happened frequently to you (that is to say, more than a few times), does it have to do with a documentation problem?

THIS QUESTION ONLY APPLIES IF ANY RESPONSE TO QUESTIONS 1-9 WAS 3 OR MORE (A FEW TIMES OR MORE)

Yes, very much so **4**

Yes, a lot **3**

Yes, a little **2**

No, nothing [to do with it] **1**

Don’t know **0**

1. Considering everything we just talked about, for those things that happened frequently to you (that is to say, more than a few times), does it have to do with your skin color?

THIS QUESTION ONLY APPLIES IF ANY RESPONSE TO QUESTIONS 1-9 WAS 3 OR MORE (A FEW TIMES OR MORE)

Yes, very much so **4**

Yes, a lot **3**

Yes, a little **2**

No, nothing [to do with it] **1**

Don’t know **0**

1. Considering everything we just talked about, for those things that happened frequently to you (that is to say, more than a few times), does it have to do with your origin?

THIS QUESTION ONLY APPLIES IF ANY RESPONSE TO QUESTIONS 1-9 WAS 3 OR MORE (A FEW TIMES OR MORE)

Yes, very much so **4**

Yes, a lot **3**

Yes, a little **2**

No, nothing [to do with it] **1**

Don’t know **0**
